# Simplified Technique for Sealing Corneal Perforations Using a Fibrin Glue-Assisted Amniotic Membrane Transplant-Plug

**DOI:** 10.1155/2014/351534

**Published:** 2014-06-18

**Authors:** Selcuk Kara, Sedat Arikan, Ismail Ersan, Arzu Taskiran Comez

**Affiliations:** Department of Ophthalmology, Canakkale Onsekiz Mart University, School of Medicine, Canakkale, Turkey

## Abstract

*Purpose*. To describe a surgical technique using amniotic membrane transplant (AMT) with fibrin glue (FG) for treating smaller corneal perforations more practically and appropriately filling the defect. *Method*. A patient with noninfectious central corneal perforation, in 1 mm in diameter, was treated with FG-assisted AMT-plug. An AMT was folded in on itself twice by using FG then a small piece of this FG-AMT mixture was cut to maintain an appropriate plug for the site of the corneal perforation. The FG-assisted AMT-plug was placed in the perforation area by using FG. An amniotic membrane patch was placed over the plug, which was then secured by a bandage contact lens. *Result*. Surgery to restore corneal stromal thickness without recurrence of perforation. *Conclusion*. The FG-assisted AMT-plug allowed a successful repair of 1 mm in diameter corneal perforation. This technique was easily performed, thus seeming to be a good alternative to treat corneal perforations with restoring corneal thickness.

## 1. Introduction

Corneal perforations presenting as a result of infection, inflammation, or trauma are ophthalmic emergencies that urgently require appropriate intervention. Management of the perforation depends on the size of the defect, the underlying disease, the surgeon's experience, and the presence of amniotic membrane transplant (AMT) or donor cornea. Medical treatment alone is effective for small perforations (<1 mm); bandage contact lenses can also be used successfully for perforations of this size [1, 2]. However, larger defects (>2 mm in diameter) require surgical procedures, including tissue adhesives, AMT, and corneal transplantation [3–5]. Although the tissue adhesives cyanoacrylate and fibrin glue are widely used in smaller corneal perforations, they cannot be sustained in the defect site as a filling material [6]. The success rate decreases to 37% with cyanoacrylate application alone in corneal perforations associated with herpetic keratitis [7].

AMT, which includes growth factors, neurotrophins, and cytokines, has been useful in the treatment of ulcerative corneal diseases [8]. Furthermore, multilayer application of AMT has also been used to restore corneal stromal thickness of perforations up to 3 mm in diameter, though Rodríguez-Ares et al. reported that the success rate decreased to 40% in perforations of 1.5 mm or larger [4, 9]. Kim and Park reported on a way to eliminate the limitations of multilayer AMT [10]. They maintained a thick single piece of 5- or 7-ply AMT by using fibrin glue (FG) between the sheets, a method called “augmented AMT,” and successfully treated corneal perforations in a range of 2–5 mm in diameter. Although this technique is very useful for larger corneal perforations, it is relatively difficult to prepare FG-assisted multilayer AMT and there is no need for the manipulation of separate pieces of AMT in small perforations. We aimed to modify this technique to treat smaller corneal perforations more practically and appropriately fill the defect by providing a mechanical scaffold.

In the current case, we present an intervention of FG-assisted AMT-plug for a corneal perforation of 1 mm in diameter and evaluate the corneal stroma stability until keratoplasty.

## 2. Case

A 76-year-old male patient was referred to our clinic with epiphora and vision decrease in his left eye. He had previously undergone a cataract surgery and had received an intraocular lens two years earlier. His visual acuity had been hand motion for two months. On examination, a 1 mm in diameter central corneal perforation with aqueous leakage and collapse of anterior chamber due to herpes simplex infection was identified. The iris was not prolapsed and there was a decreased corneal sensation in the eye of corneal perforation. Following topical anesthetic instillation we performed a protective surgery using FG-assisted AMT-plug to maintain an intact cornea before keratoplasty. On the first postoperative day, we found that the cornea had sealed and the anterior chamber had deepened without signs of inflammation. On follow-up examination, the anterior chamber was secured and a Seidel test showed a negative finding. 4 weeks later, we found the AMT-plug dissolved. During the 5-month follow-up period of this patient after surgery and before the keratoplasty, the corneal integrity was stable and there was no recurrence of the epithelial defect.

## 3. Surgical Technique

**Figure 1:**
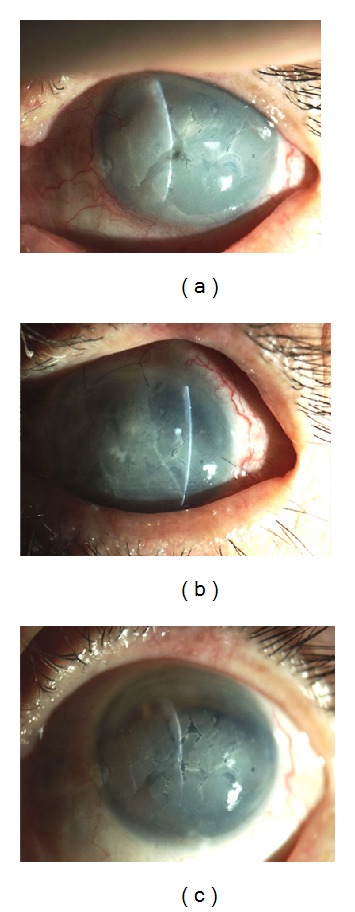
Healing of a central corneal perforation after performing FG-assisted AMT–plug. (a) A preoperative photograph of corneal perforation due to noninfectious corneal ulcer. (b) A postoperative photograph of FG-assisted AMT–plug in the defect with amniotic membrane patch overlaid, 3 days after application. (c) A stable corneal scar with nearly normal thickness and a smooth corneal surface is seen 3 months postoperatively.

**Figure 2:**
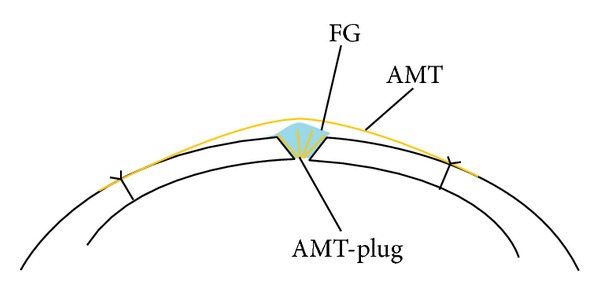
Scheme of the technique. The FG-assisted AMT-plug is in the defect with a new FG to stabilize it and amniotic membrane patch overlaid. AMT, amniotic membrane transplant; FG, fibrin glue.

Under local anesthesia, 1.8% sodium hyaluronate was used to allow an adequate anterior chamber depth without passage of viscoelastic material through the corneal opening. The perforation was dried with a microeye sponge. A 3 × 3 mm amniotic membrane transplant (AMT) was folded in on itself twice by using FG (Tisseel Lyo, Baxter, Vienna, Austria) to paste each part of AMT-fold to the other. One drop of each component of FG was applied to the AMT and right after that AMT folding was performed. We waited for several minutes for the fibrin clot to form and then cut a small piece of this fibrin glue-AMT mixture to maintain an appropriate plug for the site of the corneal perforation. We gently placed the FG-assisted AMT-plug in the perforation area and simultaneously applied FG on the plug and waited with a light pressure by a forceps until the fibrin clot was formed. We then sutured a double layer of AMT, epithelium side up, with 10/0 nylon to cover the FG-assisted AMT-plug. After that a bandage contact lens was used for 4 weeks, and topical antibiotic moxifloxacin (Vigamox, Alcon, TX, USA) was prescribed for use hourly in the first day and four times a day for the next 2 weeks (Figures 1 and 2).

## 4. Discussion

Cyanoacrylate glue and fibrin glue are easy to use and applied for the treatment of corneal perforation [11, 12]. The Leahy et al. reported that only 32% of 44 eyes with perforations and descemetoceles required no further treatment after application of cyanoacrylate tissue adhesive; however, multiple reapplications are not recommended because this will enlarge the defect [13]. Additionally, both cyanoacrylate and fibrin glue tissue adhesives are not stable at the site of defect area for a long time. Especially fibrin glue degrades faster [12]. At last, a corneal transplantation had to be performed in 45% of the eyes after applying tissue adhesives [13]. So, the treatment of corneal perforations may be much attentive with a filling scaffold material, which constructs the structure of defect site and maintains sufficient time for an adequate penetrating keratoplasty. AMT has been used to treat ocular surface epithelial disorders, to restore corneal stromal thickness and to treat small-sized corneal perforations [9, 14]. AMT and scleral patches, if it is hard to maintain AMT, are very useful to avoid an urgent penetrating keratoplasty, which has a limited success rate in acute cases [15, 16]. Although the scleral patch may be used for the same purpose, AMT provides good care of corneal defect because of its anti-inflammatory and antifibrotic properties and because it fills the defect site as a graft [8].

AMT patches have been used for corneal perforations up to 3 mm in diameter with a multilayered technique, though Rodríquez-Ares et al. reported that AMT was effective for perforations ≤1.5 mm in diameter [4]. Hick et al. increased the success rate of that technique by using fibrin glue instead of sutures [17]. Recently, Kim and Park also reported that fibrin glue-assisted augmented AM was effective for corneal perforations of 2–5 mm in diameter. They used 5- or 7-ply augmented pieces of AM that were constructed by applying fibrin glue to each sheet of AM to fill the perforated site [10].

Although multilayered amniotic membrane grafting and fibrin glue-assisted augmented AM are useful techniques, preparation and application of the AM may become difficult when treating smaller corneal perforations. We describe a modified method of augmented FG-AMT to achieve an adjustable plug for the perforation site. We termed it an “FG-assisted AMT-plug.” This method provides an easier and more practical method of AMT-plug preparation for sufficient repair of the corneal perforation. Also, this solid and biocompatible plug material has potential to promote corneal epithelial healing and to provide mechanical scaffold. After the surgical procedure the cornea was covered with a single layer of AMT and a bandage contact lens to prevent the mechanical effect on the AMT-plug caused by the eyelids and hydration of the AMT-plug by tears.

In conclusion, FG-assisted AMT-plug seems to be an effective and easier alternative for the treatment of acute corneal perforations smaller than 3 mm in diameter. This modified method may provide a shorter repair time for perforations and sufficient time for awaiting donor corneas for keratoplasty.
